# On Variations of Pitch in Percussion and Respiratory Sounds, &c.

**Published:** 1853-02

**Authors:** 


### EDITORIAL.

*On Variations of Pitch in Percussion and Respiratory Sounds, and their Application to Physical Diagnosis.* By AUSTIN FLINT, M.D., of Buffalo.—Being the Essay to which a Prize was awarded by the American Medical Association, at the Session of 1852.

WHATEVER is calculated to render our appreciation of physical signs more easy and certain, constitutes a permanently valuable addition to the science and literature of our Profession. Dr. FLINT, in the essay, the title of which we have given above, has called the attention of the Profession to the variations of pitch that accompany the healthy and morbid sounds elicited by percussion and auscultation. The word *pitch* is used by the writer in the same sense as it is by musicians; viz., to denote the particular tone or key of the sounds listened to. It is true, as Dr. FLINT observes, that most writers on physical exploration have omitted all mention of this quality of sound, and yet there is little doubt but that all experienced auscultators have recognised it as a part of the sound, without naming it as a distinct element.

In reference to the application of variations in pitch to the sounds elicited by percussion, the writer says:—" An elevation of pitch always accompanies diminution of resonance, in consequence of pulmonary consolidation. In other words, dullness of resonance is never present without the *pitch* being raised." " A practical advantage of attention to pitch," he continues, " consists, thus, in its confirming the existence of dullness. It furnishes additional evidence thereof, and adds positiveness to the conclusion, when the diminution of resonance might not be with certainty determinable." Again, he says:—" Another practical advantage, certainly equal to, if not greater than the foregoing, is derived from the fact that a distinct disparity of pitch may be apparent, in some instances, when a disparity in the amount of resonance is inappreciable." This last advantage was distinctly stated by Dr. Bowditch in his *Young Stethoscopist*, published several years since. It is also claimed that differences in pitch, being more readily appreciated than those of simple dullness, attention to this quality will render percussion over the more thickly covered parts of the chest, such as the mammary and scapular regions, more valuable.

In the second section of the essay, the writer details the variations of pitch perceived in connection with the natural tracheal, bronchial, and pulmonary sounds. Two distinct sounds are heard while listening over the trachea and bronchia—the one accompanying inspiration, the other expiration. The pitch accompanying both is high, though it varies much in different persons. It is stated to be higher in expiration than in inspiration. The natural pulmonary sound constitutes the "*vesicular respiration*," or the *respiratory murmur*, as it is now commonly called. This sound chiefly accompanies the act of inspiration; and, when it is continued to an appreciable degree in expiration, it is strictly continuous, *i. e.*, giving no interval between inspiration and expiration—which is not the case in the tracheal and bronchial sounds. The pitch of the vesicular or respiratory murmur is low, when compared with the bronchial sounds; and that part of it accompanying expiration is, in a healthy state of the lung, *lower* than in inspiration. Dr. FLINT also notes the important fact, that in fifteen apparently healthy persons examined; the pitch was "audibly higher at the summit of the right, than of the left side of the chest," in eleven—the remaining four exhibiting no difference. His observations in regard to the variations of pitch that accompany the more important sounds developed by pulmonary disease, have led him to the following important conclusions:—

"1. In the second stage of pneumonitis, the inspiratory sound is high in pitch, followed by an expiratory sound, which is frequently, if not generally, higher in pitch than the sound of inspiration; these traits being found in conjunction with more or less of the other characters which belong to the bronchial respiration.

"2. In cases of small tuberculous deposit, or incipient phthisis, the most striking modification of the respiratory sound is the elevation of pitch. This elevation of pitch is an important element of what is generally known as the *rude*, *rough*, or *harsh* respiration. If an expiratory sound be appreciable under these circumstances, it may be as high, or higher in pitch than the sound of inspiration, and the variation of pitch in the former is greater, inasmuch as the pitch of expiration in the normal murmur is lower than that of inspiration. Elevation of the pitch of expiration, therefore, may be found to be valuable as a sign of incipient phthisis, in some cases in which the variation in inspiration is not marked.

"3. If the tuberculous deposit be more abundant, the pitch of respiration is in a more marked degree elevated. The expiratory sound, if appreciable, will be more likely to be as high, or higher in pitch than the sound of inspiration. More or less of the other characters of the bronchial respiration are at the sametime present.

"4. In pleurisy with effusion, the pitch of respiratory sound is elevated, in conjunction with more or less the characters of the bronchial respiration, over the parts of the chest lying above the compressed lung. In cases of large effusion, after its complete removal by absorption, the affected side may continue to present a variation in pitch, the symmetry of the two sides being permanently impaired, in this respect, after the vesicular quality of respiration is regained.

"5. In cases in which tubercle has advanced to the stage of excavation, the site of a cavity of considerable size is indicated by a blowing sound, low in pitch, with an expiratory sound (if appreciable) lower in pitch than the sound of inspiration. These traits constitute the elements of the cavernous respiration, and the cavernous respiration is the most constant and reliable of the signs of an excavation.

"If the cavity be very large, or there are several cavities, the respiration may be modified to such an extent that, on immediate auscultation, over the whole summit of the chest, it may present the cavernous characters. This may be the case, while dullness on percussion shows the existence of more or less solidification in connection with the cavities. The coexistence of relative dullness on percussion, and a low-pitched blowing respiration, denotes the predominance of excavation.

"The cavernous respiration may also be present in cases of excavation from circumscribed gangrene, and in pneumothorax with perforation.

"6. In arrested phthisis, the traces of the disease may be manifested by a permanent variation in the pitch of respiration, in connection with more or less dullness on percussion at the summit of the chest on either side."

These propositions are worthy of careful study; and we shall take the earliest opportunity, that other imperative engagements will afford, for giving them a more extended and practical examination. The Essay is accompanied by an appendix, containing a brief record of observations on more than forty cases of pulmonary disease, presenting many points of practical interest, and well illustrating the commendable industry and zeal of the author.

